# Laparoscopic resection of a primary diaphragmatic schwannoma: a case report and literature review

**DOI:** 10.1186/s12893-020-00963-w

**Published:** 2020-11-19

**Authors:** Ayako Kamiya, Yukinori Yamagata, Hiroshi Yoshida, Kenichi Ishizu, Takeyuki Wada, Tsutomu Hayashi, Sho Otsuki, Takaki Yoshikawa, Hitoshi Katai

**Affiliations:** 1 Department of Gastric Surgery, National Cancer Center Hospital, 5-1-1 Tsukiji, Chuo-ku, Tokyo, 104-0045 Japan; 2 Division of Pathology and Clinical Laboratories, National Cancer Center Hospital, 5-1-1 Tsukiji, Chuo-ku, Tokyo, 104-0045 Japan

**Keywords:** Schwannoma, Diaphragm, Laparoscopic resection

## Abstract

**Background:**

Schwannomas are nerve sheath tumors that commonly originate from the stomach and small intestine. A primary schwannoma of the diaphragm is rare and does not show any symptoms until it grows to a certain size. Hence, it is extremely rare that it was found at a size that allowed resection under videoscopic surgery.

**Case presentation:**

A 77-year-old woman was referred to our department for surgical treatment of a tumor located near the gastric fornix. She underwent a routine esophagogastroduodenoscopy 2 years and 7 months prior to the referral. It was suspected that she had a submucosal tumor measuring 10 mm, located in the fornix, and was then referred to her previous physician. During her follow-up, endoscopic ultrasonography (EUS) revealed that the cystic structure had continued to grow toward the gastric wall, and she was then referred to the endoscopy division of our hospital. She continued to be followed-up, and it was noted that the tumor was gradually increasing in size. Therefore, she requested surgical resection, and was finally referred to our division. Since the tumor was rather small, we planned a laparoscopic surgery. An initial examination during the operation revealed that the tumor was located on the left diaphragm. Since the tumor was relatively small and visibility was good, we decided to continue with the laparoscopic surgery. Partial diaphragmectomy with complete inclusion of the tumor was performed, and the defect of the diaphragm was directly closed by a running suture. Pathological examination revealed a benign schwannoma that had originated from the diaphragm. To support our findings, we also reviewed the scientific literature on diaphragmatic schwannoma cases reported up to April 2020.

**Conclusions:**

In this extremely rare case, we successfully resected the diaphragmatic schwannoma using laparoscopic surgery.

## Background

Schwannomas are nerve sheath tumors that commonly originate from the extremities, head, neck, and the posterior mediastinum [1]. The most common sites for schwannomas are the stomach and small intestine, and they rarely occur in the liver, pancreas, kidney, brain, heart, adrenal gland, retroperitoneum, and diaphragm [2]. Primary schwannoma of the diaphragm is rare, and the patients do not show any symptoms until it grows to a certain size. Moreover, it is extremely rare that a schwannoma grows to a size that allows resection using videoscopic surgery.

Here, we report an exceedingly rare case wherein a diaphragmatic schwannoma was successfully resected using laparoscopic surgery.

## Case presentation

**Fig. 1:**
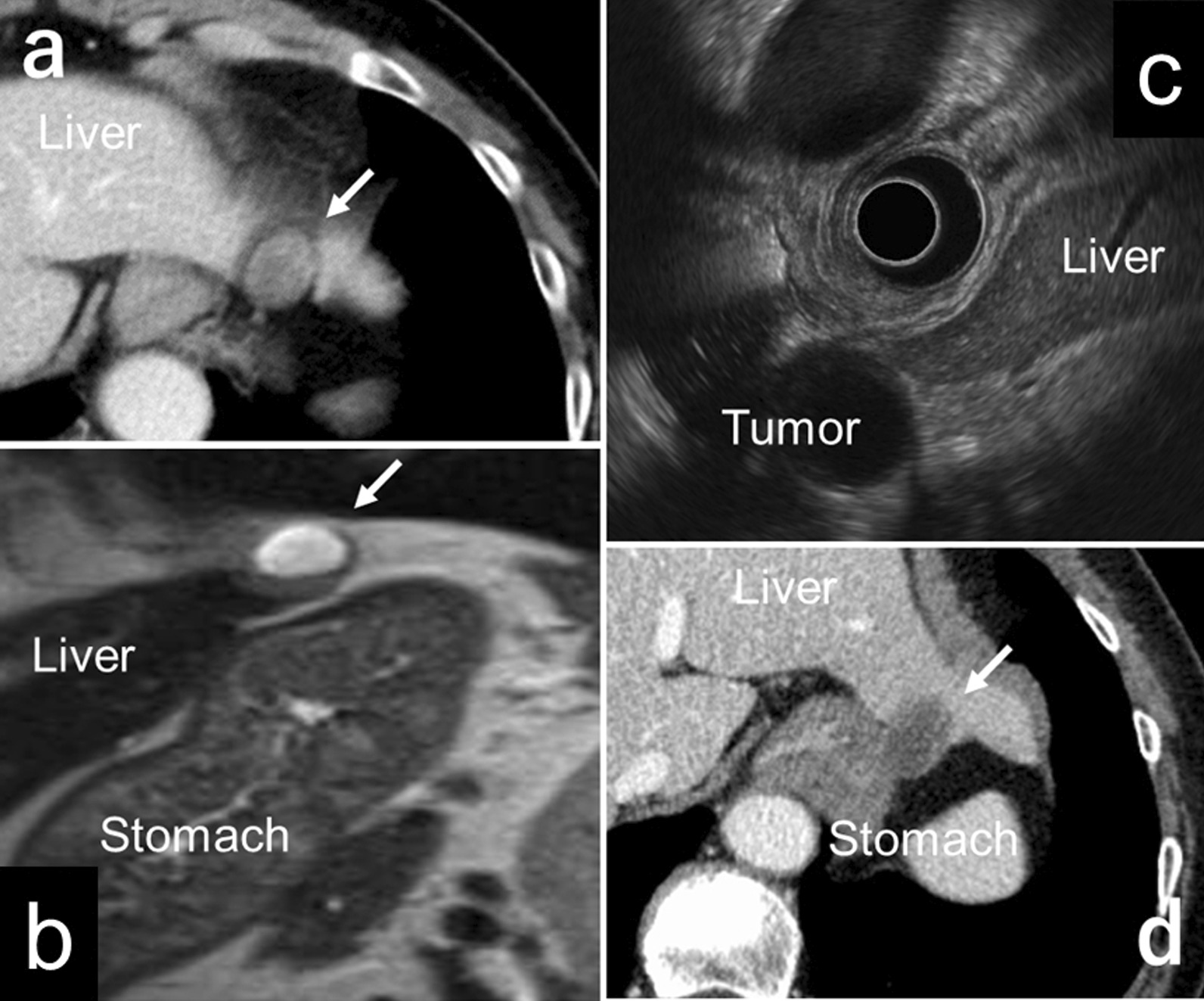
Preoperative imagings. **a** Computed tomography on admission to previous hospital showed that there was a low density, round mass, measuring approximately 20 mm in diameter, located between the upper gastric body and left lateral segment. **b** T2-weighted magnetic resonance imaging on admission to previous hospital showed a high signal intensity mass, which seemed to be distinct from the stomach. **c** The latest endoscopic ultrasonography suggested that the tumor was distinct from the stomach and the liver. **d** The latest computed tomography showed an unclear part of the boundary between the tumor and the gastric wall

A 77-year-old woman was referred to our department after she requested surgical treatment for a tumor located near the gastric fornix. She underwent a preventive esophagogastroduodenoscopy 2 years and 7 months prior to the referral. The examination had revealed a submucosal tumor-like lesion in the fornix, which measured 10 mm. After this diagnosis, she was referred to her previous physician. Contrast enhanced computed tomography (CT) revealed a low density, round mass, measuring approximately 20 mm that was located between the upper stomach and left lateral segment of the liver (Fig. 1a). Additionally, magnetic resonance imaging (MRI) revealed that the mass showed a high signal intensity on T2-weighted imaging and seemed to be distinct from the stomach (Fig. 1b). It was determined that the tumor would require careful follow-up. A follow-up MRI was performed 5 months later, and it showed slight growth of the tumor. An endoscopic ultrasonography (EUS) was also performed, and it revealed a cystic structure continuous with the gastric wall. Contrary to the previous CT and MRI results that suggested the presentation of an extra-gastric tumor, the EUS results suggested that a gastrointestinal stromal tumor (GIST) could not be ruled out. Owing to the conflicting results, the patient was referred to the endoscopy division of our hospital two years ago for detailed examination. On admission, abnormal symptoms such as fever, anemia, and jaundice were not observed, and her performance status was good (Eastern Cooperative Oncology Group score of 0). She had a history of Sjögren syndrome but had no history of other systemic diseases such as diabetes and hypertension. She had no history of smoking and alcohol abuse. Additionally, she had no family history of malignant diseases. Laboratory studies were within normal range. The EUS was re-examined, and it was concluded that the cystic tumor with solid components did not to originate from either the liver or the stomach. Fine-needle aspiration biopsy was not performed considering the risk of tumor dissemination (according to Japanese Clinical Practice Guidelines for GIST, 3rd edition, it is contraindicated to perform EUS-FNA for extramurally grown submucosal tumor [3]). The differential diagnoses considered for the tumor were bronchogenic cyst, epidermoid cyst, lymph node with cystic degeneration, and neurogenic tumor. Since a few malignant findings were observed, regular follow-ups were recommended for the patient. These examinations indicated that there was gradual growth in the size of the tumor. A follow-up EUS performed 1 year and 6 months after admission revealed that the cystic tumor had grown to 25 mm in diameter, and there seemed to be no continuity between the tumor and the stomach (Fig. 1c). However, the CT scan showed an unclear part of the boundary between the tumor and the gastric wall (Fig. 1d). We could not completely exclude the possibility of GIST, and we explained the results to the patient. She requested surgical resection, following which we planned for a surgery at our division.

**Fig. 2:**
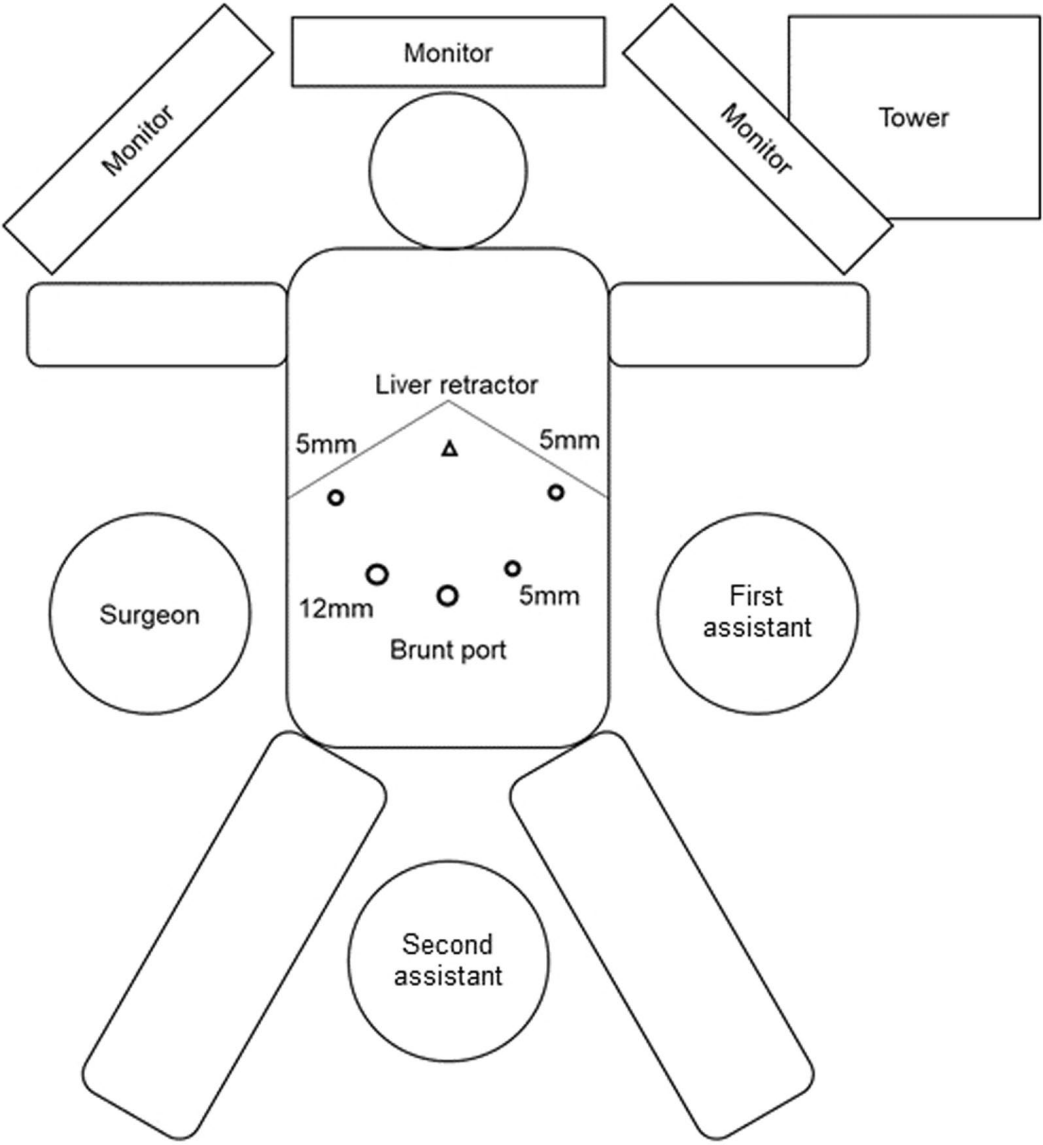
The location of trocars and retractor. The patient was placed in the supine position with open legs, brunt port was inserted from the umbilicus, a 12 mm trocar and three 5 mm trocars were placed in the reverse trapezoid position in the upper abdomen, and the liver retractor was placed in epigastrium

**Fig. 3:**
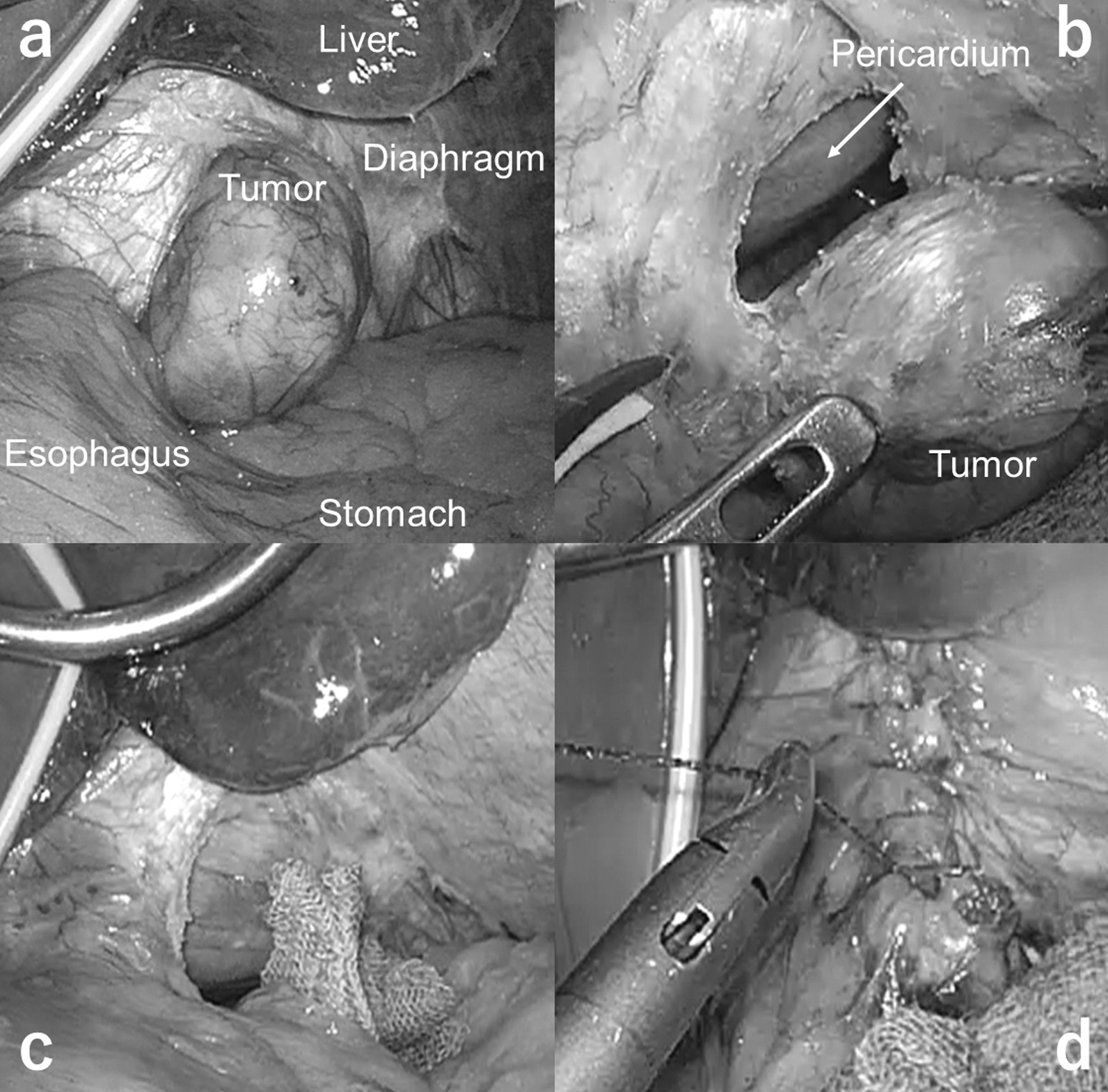
Intraoperative findings. **a** An exploratory laparoscopy revealed a firm, completely encapsulated mass located on the left diaphragm. **b** Partial diaphragmectomy was performed using laparoscopic coagulation shears. **c** Partial diaphragmectomy including the tumor was completed. **d** The defect of the diaphragm was directly closed with an absorbable suture

Since the tumor was rather small (less than 3 cm in diameter), we decided to resect the tumor using laparoscopic approach. The tumor was located close to the upper stomach, and we planned to place the trocars using the same arrangement as in upper gastrointestinal surgery. The patient was placed in the supine position with legs apart; the brunt port was inserted from the umbilicus; a 12 mm trocar and three 5 mm trocars were placed in the reverse trapezoid position in the upper abdomen; and the Nathanson’s retractor (HEIWA MEDICAL INSTRUMENTS Co., Ltd. Bofu, Yamaguchi, Japan) was placed in the epigastrium as the liver retractor (Fig. 2). An initial laparoscopic examination revealed a firm, completely encapsulated mass located on the left of the diaphragm (Fig. 3a). Since the tumor was relatively small and visibility was good, we decided to continue with the laparoscopic approach. Partial diaphragmectomy with complete inclusion of the tumor was performed using laparoscopic coagulation shears (Fig. 3b, c). The tumor was packed in a plastic bag and then extracted via the umbilical incision. The defect of the diaphragm was directly closed by a running suture using the 3-0 V-Loc™ absorbable suture (Medtronic plc, Minneapolis, MS, USA) (Fig. 3d). The total operating time was 59 min. The intraoperative blood loss was negligible. A video recording of the surgical procedure has been provided in the Additional file 1.

**Fig. 4:**
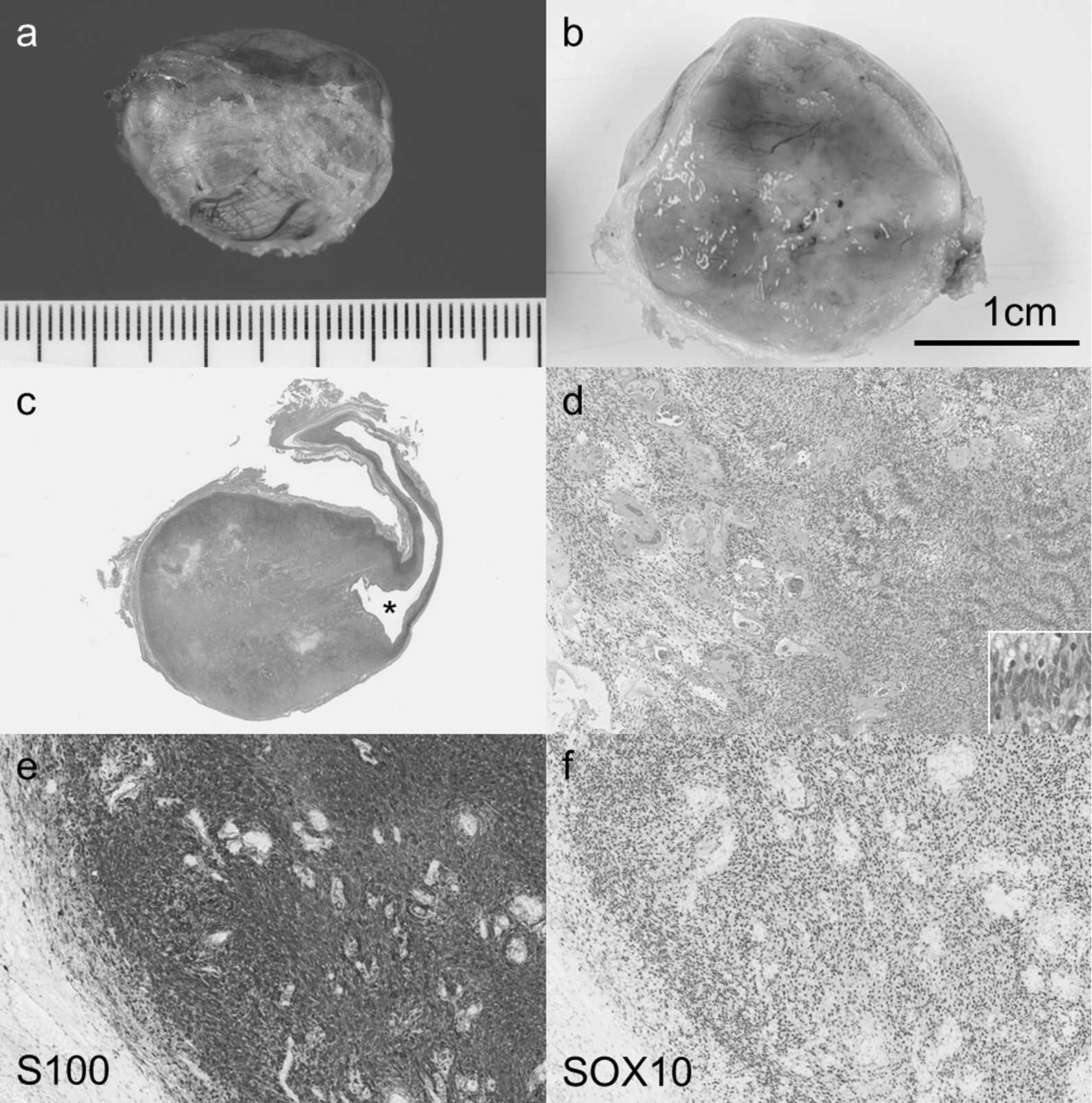
Pathological findings of the schwannoma of the diaphragm. **a** The tumor is well encapsulated and measures 2.5 × 2.0 × 1.8 cm. **b** On the cut section, the tumor shows tan or yellow solid appearance. **c** In panoramic view, the tumor can be seen surrounded by a pink fibrous capsule and shows cystic abnormalities (*). **d** At low magnification, the tumor shows the pattern of alternating Antoni type A (right side) and type B areas (left side). In the Antoni type A area, spindle cells with bland twisted nuclei show nuclear palisading (inset). **e** Immunohistochemical staining shows that the S100 protein was diffusely expressed in the tumor cells. **f** Immunohistochemical staining shows that the SOX10 protein was diffusely expressed in the tumor cells

The tumor measured 2.5 × 2.0 × 1.8 cm and was well encapsulated (Fig. 4a). The cut section of the tumor showed a tan or yellow solid component (Fig. 4b) and cystic abnormalities. On microscopic examination, it was found that the tumor was surrounded by a pink fibrous capsule with a cystic area (Fig. 4c). Under low magnification power, the tumor showed a pattern of alternating highly cellular Antoni type A and less cellular Antoni type B areas (Fig. 4d). The tumor was composed of spindle cells with bland, twisted nuclei and indistinct cytoplasmic border arranged in short bundles or interlacing fascicles (Fig. 4d). In the Antoni type A area, the spindle cells showed nuclear palisading. Upon immunohistochemical analysis, the tumor cells showed diffuse positivity for both the S100 and SOX10 proteins (Fig. 4e, f). It was concluded that the tumor was a benign diaphragmatic schwannnoma.

There were no postoperative complications. The patient was discharged on the fifth postoperative day and is doing well 4 months after the surgery.

## Discussion

This report describes a rare case of primary schwannoma of the left diaphragm. Schwannomas are nerve sheath tumors that commonly originate from the extremities, head, neck, and the posterior mediastinum [1]. The most common sites of a schwannoma are the stomach and small intestine, and primary schwannoma of the diaphragm is rare [2]. We searched all articles on schwannomas in the PubMed database, published up to April 2020 using the following keywords: ((((schwannoma) OR (neurinoma)) OR (neurilemoma)) AND (diaphragm)) AND (English [Language]). We acquired 36 records, from which 20 articles were excluded because of obvious irrelevance, narrowing the list down to 16 case reports. Finally, including our case, 17 cases of primary diaphragmatic schwannoma were collected, and their summary is provided in Table 1 [2, 4–18].

**Table 1:** Summary of diaphragmatic schwannoma cases

Case	Author	Year	Age	Gender (Male or female)	Side (Right or left)	Symptoms				Size (mm)	Surgical approach		Benign or malignant	Prognosis
						Pain	Dyspnea	Cough	Clubbing		Thoracic or abdominal	Open or videoscopic		
1	Weisel [4]	1956	55	F	L	+	+	−	−	230	T	O	Unknown	Unknown
2	Trivedi [5]	1958	5	F	L	−	−	−	+	75	T	O	Unknown	Unknown
3	Sarot [6]	1969	65	M	L−	−	−	−	−	95	T	O	Unknown	Unknown
4	McHenry [7]	1988	45	F	L	+	−	−	−	35	A	O	B	Not mentioned
5	McClenathan [8]	1989	46	F	R	−	−	−	−	100	T	O	B	4 years, Disease-free
6	Lee [9]	1991	36	F	R	+	−	−	−	100	T	O	M	Not mentioned
7	Urschel [10]	1994	84	F	R	−	−	+	−	85	T	O	B	Not mentioned
8	Koyama [11]	1996	38	F	L	−	−−	−	−	51	A	O	B	Not mentioned
9	Ikegami [12]	2004	47	M	R	−	−	−	−	50	A	O	B	Not mentioned
10	Kumbasar [13]	2004	48	F	R	+	−	−	−	330	T	O	M	32 months, disease-free
11	Ohba [14]	2008	50	F	R	−	−	−	−	104	T	O	B	6 months, disease-free
12	Chang [15]	2012	38	F	L	+	+	−	−	140	T	O	B	1 year, disease free
13	Hobbs [16]	2012	13	F	R	+	−	−	−	120	A	O	B	6 months, disease-free
14	Liu [17]	2013	46	M	R	−	−	−	−	30	A	V	B	2 years, disease-free
15	Bhattar [2]	2018	39	M	L	+	−	−	−	100	A	O	B	Not mentioned
16	Abraham [18]	2019	68	F	L	−	−	+	−	20	T	V	B	1 year, disease-free
17	Present case	2020	77	F	L	−	−	−	−	24	A	V	B	4 months, disease-free

The median age of these 17 patients was 47 years old (range 5–84). Diaphragmatic schwannoma can occur in patients across a wide range of age groups, affecting children to the elderly. However, it seems to occur most commonly in middle-aged individuals. Among the patient reports, 13 were female and 4 were male; women were more likely to develop the disease. We noted that 8 cases occurred on the right side of the diaphragm and 9 cases on the left side, without any other differences in presentation.

In 10 cases the patients reported symptoms and the frequency of pain was particularly high. The mean tumor size was 124 mm in cases with symptoms, and 63 mm in asymptomatic cases. Thus, the presence of symptoms seemed to be associated with tumor size.

In all 17 cases, the tumors were completely resected. In 10 cases, the tumors were resected using the transthoracic approach, and in 7 cases the resection was performed using the transabdominal approach. The mean tumor size for the transthoracic cases was 127 mm and for the transabdominal cases was 58 mm. The larger the tumor, the more challenging was the use of the transabdominal approach. In recent cases, relatively small tumors were resected under a videoscope. Selection of the type of surgical technique (laparoscopic or thoracoscopic) seemed to depend on the location of the tumor and whether the tumor protruded to the thoracic or the abdominal cavity.

Schwannomas are usually slow-growing nerve sheath tumors that are derived from the Schwann cells. Usually, these remain encapsulated and do not invade the surrounding organs, and most patients are asymptomatic while the tumor remains small [1]. They are often identified incidentally using imaging techniques such as CT, MRI, and US [1]. Advancements in diagnostic imaging in the future may lead to an increase in the number of relatively small sized diaphragmatic schwannomas that are identified as well as an increase in the number of videosurgical procedures for this tumor.

As mentioned above, schwannomas are usually benign in nature [1]. However, some schwannomas may become malignant as they grow in size. In fact, malignant cases were confirmed in 2 out of the 17 cases discussed here [9, 13]. Notably, only a few cases reported on long-term prognosis of patients, and effective treatment methods other than resection have not been studied. Further case studies are needed regarding the prognosis and the form of metastasis or recurrence of this tumor.

In this case, we were not aware that the tumor was located on the diaphragm before the surgical procedure. When we observed that the tumor was on the diaphragm in the initial laparoscopic examination, we decided to continue with laparoscopic surgery because the tumor was relatively small and visibility was good. Generally, whether a diaphragmatic tumor can be resected under a laparoscope will probably depend on the size and location of the tumor. Since the liver is located in the upper right abdomen, it is probably easier to resect a tumor on the left side of the diaphragm than on the right side. Additionally, a tumor protruding to the abdominal side is easier to resect using the abdominal approach because it can be visualized from the abdominal cavity side. After performing the diaphragmectomy using the open approach, usually the defect is closed by direct suture if it is relatively small. Alternatively, the diaphragm is repaired using an autologous or artificial material if it is too large to close directly. If the field of view and the working space are sufficiently secured, it would be possible to perform the same procedure under a laparoscope [17]. In our case, the diaphragmatic defect after tumor resection was not large. Hence, it could be easily repaired with a simple suture. The possibility of tension pneumothorax should be given due consideration when the diaphragm defect becomes large and the repair takes time [17]. In this case, it took only 15 min to complete the repair after communicating with the thoracic cavity. Moreover, the pneumoperitoneum pressure was reduced from 12 to 8 mmHg after the abdominal cavity communicated to the thoracic cavity. Therefore, the operation could be completed without any issues.

## Conclusion

We experienced an extremely rare case of diaphragmatic schwannoma. We reviewed 17 cases of diaphragmatic schwannoma cases and showed that they lacked characteristic findings and their fewer symptoms made it difficult to detect them in a certain size. However, in recent cases, it seemed that more tumors were found in relatively small size and were resected under a videoscope.

Our case was also found in a small size of 2.5 cm in diameter, and successfully achieved complete resection under laparoscopic surgery.

## Supplementary information

**Additional file 1:** A video recording of the surgical procedure. Partial diaphragmectomy was performed using laparoscopic coagulation shears. Then the defect of the diaphragm was directly closed with an absorbable suture.

## Data Availability

The data supporting the conclusions of this article have been included in this published article**.**

## Abbreviations

CT: Computed tomography
MRI: Magnetic resonance imaging
EUS: Endoscopic ultrasonography
GIST: Gastrointestinal stromal tumor

## References

[1] Cutfield SW, Wickremesekera AC, Mantamadiotis T, Kaye AH, Tan ST, Stylli SS (2019). Tumour stem cells in schwannoma: a review. J Clin Neurosci.

[2] Bhattar R, Tomar V, Dhakad DS, Agarwal N (2018). Benign diaphragmatic neurilemmoma mimicking a left adrenal cyst. Turk J Urol.

[3] Japan Society of Clinical Oncology, Japanese Gastric Cancer Association, Japanese Study Group on GIST. Japanese Clinical Practice Guidelines for Gastrointestinal Stromal Tumor (GIST). 3rd ed. Tokyo: KANEHARA & CO., LTD; 2014. p. 17–30.

[4] Weisel W, Claudon DB, Willson DM (1956). Neurilemmoma of the diaphragm. J Thorac Surg.

[5] Trivedi SA (1958). Neurilemmoma of the diaphragm causing severe hypertrophic pulmonary osteoarthropathy. Br J Tuberc Dis Chest.

[6] Sarot IA, Schwimmer D, Schechter DC (1969). Primary neurilemmoma of diaphragm. N Y State J Med.

[7] McHenry CR, Pickleman J, Winters G, Flisak ME (1988). Diaphragmatic neurilemoma. J Surg Oncol.

[8] McClenathan JH, Okada F (1989). Primary neurilemoma of the diaphragm. Ann Thorac Surg..

[9] Lee JT, Lee JD, Choe KO, Yang WI (1991). Giant malignant schwannoma of the diaphragm: CT and ultrasound findings. Yonsei Med J.

[10] Urschel JD, Antkowiak JG, Takita H (1994). Neurilemmoma of the diaphragm. J Surg Oncol.

[11] Koyama S, Araki M, Suzuki K, Fukutomi H, Maruyama T, Mun Y (1996). Primary diaphragmatic schwannoma with a typical target appearance: correlation of CT and MR imagings and histologic findings. J Gastroenterol.

[12] Ikegami T, Ezaki T, Ishida T, Kohno H, Fujihara M, Mori M (2004). Neurilemmoma of the diaphragm mimicking a liver tumor: case report. Abdom Imaging.

[13] Kumbasar U, Enon S, Osman Tokat A, Gungor A (2004). An uncommon tumor of the diaphragm malignant schwannoma. Interact Cardiovasc Thorac Surg.

[14] Ohba T, Shoji F, Kometani T, Yoshino I, Maehara Y (2008). Schwannoma in the peridiaphragm. Gen Thorac Cardiovasc Surg.

[15] Chang CY, Chang YC, Chang SC, Chen YC (2012). A huge diaphragmatic schwannoma mimicking diaphragm palsy. QJM.

[16] Hobbs DJ, McLellan J, Schlatter MG (2012). Schwannoma of the diaphragm-a pediatric case report and review of the literature. J Pediatr Surg.

[17] Liu K, Zhang M, Liang X, Cai X (2013). Laparoscopic phrenectomy for a diaphragmatic neurilemmoma. J Res Med Sci.

[18] Abraham E, Schwartz G (2019). Primary schwannoma of the diaphragm. Proc (Bayl Univ Med Cent).

